# Reciprocal Dysregulation of MiR-146b and MiR-451 Contributes in Malignant Phenotype of Follicular Thyroid Tumor

**DOI:** 10.3390/ijms21175950

**Published:** 2020-08-19

**Authors:** Margarita Knyazeva, Ekaterina Korobkina, Alexey Karizky, Maxim Sorokin, Anton Buzdin, Sergey Vorobyev, Anastasia Malek

**Affiliations:** 1 Subcellular technology Lab., N. N. Petrov National Medical Center of Oncology, 197758 Saint Petersburg, Russia; margo9793@gmail.com (M.K.); kat.korspb@gmail.com (E.K.); 2 Oncosystem Company Limited, 121205 Moscow, Russia; 3 Institute of Biomedical Systems and Biotechnologies, Peter the Great Saint. Petersburg Polytechnic University (SPbPU), 195251 Saint Petersburg, Russia; 4 Information Technologies and Programming Faculty, Information Technologies, Mechanics and Optics (ITMO) University, 197101 Saint-Petersburg, Russia; alexei_karitski@mail.ru; 5 Institute of Personalized Medicine, I.M. Sechenov First Moscow State Medical University, 119048 Moscow, Russia; sorokin@oncobox.com (M.S.); buzdin@oncobox.com (A.B.); 6 Omicsway Corporation, Walnut, CA 91789, USA; 7 Shemyakin-Ovchinnikov Institute of Bioorganic Chemistry, 117997 Moscow, Russia; 8 National Center of Clinical Morphological Diagnostics, 192283 Saint Petersburg, Russia; slvorob@gmail.com

**Keywords:** follicular thyroid cancer, follicular adenoma, miRNA, RT-qPCR, reciprocal dysregulation, invasion

## Abstract

Over the last few years, incidental thyroid nodules are being diagnosed with increasing frequency with the use of highly sensitive imaging techniques. The ultrasound thyroid gland examination, followed by the fine-needle aspiration cytology is the standard diagnostic approach. However, in cases of the follicular nature of nodules, cytological diagnosis is not enough. Analysis of miRNAs in the biopsy presents a promising approach. Increasing our knowledge of miRNA’s role in follicular carcinogenesis, and development of the appropriate the miRNA analytical technologies are required to implement miRNA-based tests in clinical practice. We used material from follicular thyroid nodes (*n*.84), grouped in accordance with their invasive properties. The invasion-associated miRNAs expression alterations were assayed. Expression data were confirmed by highly sensitive two-tailed RT-qPCR. Reciprocally dysregulated miRNAs pair concentration ratios were explored as a diagnostic parameter using receiver operation curve (ROC) analysis. A new bioinformatics method (MiRImpact) was applied to evaluate the biological significance of the observed expression alterations. Coupled experimental and computational approaches identified reciprocal dysregulation of miR-146b and miR-451 as important attributes of follicular cell malignant transformation and follicular thyroid cancer progression. Thus, evaluation of combined dysregulation of miRNAs relevant to invasion and metastasis can help to distinguish truly malignant follicular thyroid cancer from indolent follicular adenoma.

## 1. Introduction

Follicular thyroid cancer (FTC) is a variant of thyroid-derived neoplasm that preserves follicular pattern and cell differentiation. FTC differs from other types of thyroid malignancies in terms of its clinical behavior, the pathological appearance and the genetic landscape [1,2]. The most intriguing issue in the FTC management is distinguishing between truly malignant FTC and benign follicular adenoma (FA). It can sound a controversial statement, but currently no available diagnostic approaches can confidently address this question. The absolute criterion for the discrimination between FTC and FA, apparently, is a convincing local invasion or an appearance of distant metastases.

Due to absence of cellular or nuclear atypia, cytological analysis of the material obtained by fine-needle aspiration can only reveal follicular nature of the thyroid node and predict the risk of malignancy in quite broad range: 10%–40% in the case of follicular neoplasm (Bethesda IV) and 6%–28% in the case of or follicular lesion of undetermined significance (Bethesda III) [3]. Pathological examination of the tumor capsule after thyroidectomy is the only approach to confirm FTC [4]. However, identification of the capsular invasion may be not a trivial issue simply because of the impossibility of complete examination of the tumor capsule. Quality of the diagnosis depends on the number of evaluated sections, that lacks standardization [5] and gives rise for liberal choice of diagnostic algorithm. Described difficulties in the pathological evaluation of FTC leads to diagnostic discrepancies [6] and variability in therapeutic approaches [7]. Searches for genetic markers of follicular cancer were anticipated to provide reliable diagnostic criteria. It is assumed, for instance, that *RAS* point mutations and *PAX8/PPARγ* chromosomal rearrangements are the main drivers in follicular thyroid tumor development [8]. However comparative prevalence of *RAS* mutations in FTC (40%–50%) and FA (20%–40%) doesn’t allow the use of this genetic marker for the purpose of differential diagnosis [9]. Fusions of *PAX8/PPARγ* regions are found more frequently in FTC (30%–40%) than in FA (2%–13%) [9], however this difference does not allow to discriminate FTC from FA. Mutations in some other genes, such as *TERTp*, *EZH1*, *EIF1AX*, *TSHR*, *H/N/K-RAS* and *TP53*, were being explored as a potential differential markers for FTC and FA [10,11,12], but results are still not confident enough to be implemented in clinical practice.

In the context of thyroid cancer research, miRNA signatures of the thyroid nodules is being actively explored as a diagnostic criteria of overall malignancy [13], as well as diagnostic features of specific histological types of thyroid tumors: poorly differentiated thyroid cancer [14], conventional and oncolytic follicular thyroid cancer [15], specific types of papillary thyroid cancer [16] or follicular thyroid neoplasm with papillary-like nuclear features [17]. Differences of miRNA profiles in FTC and FA have been studied as well [18,19,20]. For instance, Stokowy T. and co-authors performed a meta-analysis of miRNA expression profiling data retrieved from several studies and revealed that certain data overlap. Moreover, using the so-called “ruling in malignancy” method they developed two-miRNA classifiers allowing for the distinguishing between FTC and FA, with relatively high accuracy [20,21]. However, great discrepancy between results reported by the different research groups reveal our lack of understanding regarding the role of miRNAs in follicular thyroid cancer development and progression.

In the presented study, we also attempted to develop an miRNA-based test for the discrimination between FTC and FA, while using new analytic approaches. First, in previously published investigations, preliminary selection of diagnostic miRNA candidates was traditionally performed by comparative profiling of miRNA in two groups of samples: FA and FTC [18,19,20]. Considering invasive potency as the principal discriminative criterium, we compared groups of samples with gradually changing invasive characteristics (FA < minimally invasive FTC < widely invasive FTC) and identified miRNAs with expression alterations associated with invasion. This approach was assumed to reduce the detection of random events. Second, the short length of miRNA molecules and presence of non-mature forms, make miRNA analysis by conventional reverse transcription followed by qPCR quite difficult. This issue apparently leads to an observed discrepancy of data coming from different groups. In order to improve the miRNA quantification, we used highly specific two-tailed RT-qPCR technology proposed by P. Androvic et al. Ref. [22] with certain modifications. Third, the absence of a reliable method of miRNA RT-qPCR data normalization makes it problematic to interpret the results [23]. In order to solve the data normalization issue, we explored a new parameter: the concentration ratio of two miRNAs with reciprocal characteristics of invasion-associated expression changes. We described this approach previously [24], here we have developed and used a programmatic algorithm for the selection of the best miRNA pairs. Additionally, gene ontology (GO) functional annotation and pathway activation analysis approaches [25] were applied to explore the functional significance of reciprocal miRNA pair alterations to invasion and metastasis.

Our results revealed that reciprocal dysregulation of miR-146b and miR-451a is associated with an increased invasive potency of follicular thyroid tumor. The ratio of miR-146b and miR-451a concentrations in a bio-samples, therefore, might present a promising marker for discrimination between FTC and FA.

## 2. Results

### 2.1. Selection of Candidate MiRNAs

In order to select miRNAs associated with invasive properties of follicular thyroid tissue, we collected formalin fixed paraffin embedded (FFPE) samples of the thyroid nodules after thyroidectomy and independent evaluation by two pathologists confirming them as either follicular adenoma (FA), minimally invasive follicular thyroid cancer (miFTC) or widely invasive follicular thyroid cancer (wiFTC). After RNA isolation, samples with a concentration of 0.5–1 μg/mkl and a 260/280 ratio higher than 1.8 were combined in equivalent quantities in three pools, representing different histological diagnoses: FA (*n*.10), miFTC (*n*.10) and wiFTC (*n*.10). Expression levels of 170 miRNAs were analysed by miRCURY LNA miRNA PCR Panel. The interplate amplification rates discrepancy was corrected with interplate calibrators. A value of cycle threshold (Ct) higher than 38 was considered as background and excluded from analysis. Results were normalized to the global Ct mean (28.2). In total there were 110 miRNAs detected in all three pooled samples. The expression of 30 miRNAs was gradually increased in a row “FA < miFTC < wiFTC”, while expression of eight miRNAs was gradually decreased in a row of samples “FA > miFTC > wiFTC”. Results are presented as Supplementary Data 1. Thus, expression and functional activity of these molecules might be associated with the invasive properties of follicular cells.

**Figure 1:**
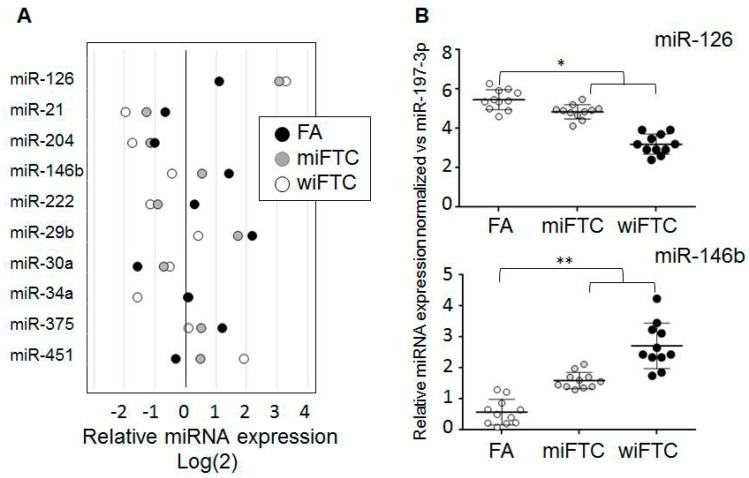
Profiling of 170 miRNA in samples of follicular adenoma (FA), miFTC and wiFTC. (**A**) Selected miRNAs with gradually altered expression in row of follicular adenoma (FA)—minimally invasive FTC (miFTC)—widely invasive follicular thyroid cancer (FTC) (wiFTC). RT-qPCR was performed with miRCURY LNA miRNA Focus PCR Panel using three pooled samples. Results RT-qPCR were normalized to global Ct mean and Log2 transformed. (**B**) Results of miR-126 and mir146b analyses by RT-qPCR in individual samples of FA, miFTC and wiFTC. Results RT-qPCR were normalized to miR-197-3p. Statistical significance was calculated using the one tailed Mann-Whitney U test and is indicated by * (*p* < 0.05) and ** (*p* < 0.005).

We selected 10 miRNAs (miR-21, miR-29b, miR-20a, miR-146b, miR-204, miR-451, miR-375, miR-126, miR-222 and miR-34a) for further evaluation based on the following criteria: degree of invasion-associated expression alterations observed in our assay, involvement in the regulation of invasive behavior of cancer cells confirmed by other studies and structure of mature miRNA that allowed confident design of a two-tailed RT-qPCR analytic system. Expression profiles of miRNAs selected are shown in Figure 1A. The expression alterations of the selected miRNAs were observed in a range of 1.5–2 fold only, however these alterations were obviously associated with invasive properties of thyroid nodes.

In order to investigate if results of the pooled samples analysis did reflect miRNA expression profiles of the whole groups, we assayed two miRNAs (miRNA-126 and miRNA-146b) in individual samples of each group: FA (*n*.10), miFTC (*n*.10) and wiFTC (*n*.10). To be confident with the normalization approach, results were normalized using two independent references: U6 small nuclear RNA (snRNA) and hsa-miRNA-197-3p [26] and grouped in accordance with histological diagnosis. After both normalization methods, the expression of miRNA-126 and miRNA-146b in the groups obviously differed. However, a statistically significant difference was observed when comparison of FA vs. the group composed from miFTC and wiFTC for both miRNAs was tested. An example of the results (normalized vs. U6 snRNA) is presented in Figure 1B.

### 2.2. Construction and Validation of Two-Tailed RT-qPCR MiRNA Analysis Systems

According to P. Androvic et al. [22,27], we designed ten systems for the analysis of selected miRNAs using a two-tailed reverse transcription primer and two miRNA-specific PCR primers. We modified the original method by using fluorescent (FAM)-labelled probe for detecting the real-time amplification. Analytic properties of new systems, further designed as Spec-TT (miR-specific two-tailed RT), were evaluated using synthetic analogues (mimics) of the corresponding miRNAs.

**Figure 2:**
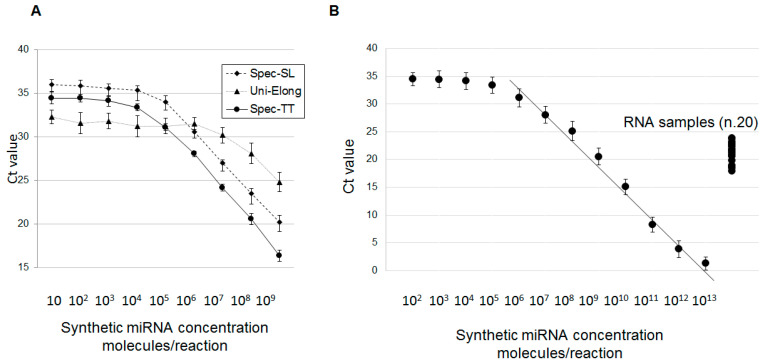
Validation of RT-qPCR system for miRNA analysis. (**A**) Efficacy of Spec-SL, Uni-Elong and Spec-TT systems for RT-qPCR analysis of miR-451 tested with different concentrations of synthetic mimic. All reactions were performed in triplicate. (**B**) Spec-TT systems for RT-qPCR analysis of miR-451 was tested in a broad range of synthetic mimic concentrations (12^2^–10^13^ molecules per reaction) and with 20 samples of RNA isolated from randomly selected thyroid node specimens (1 μm per reaction).

First, we wanted to compare the analytic efficacy of the new system with other methods. The method of reverse transcription (RT) with the miRNA-specific stem-looped primer (miR-specific stem-looped RT, further designed as Spec-SL) followed by qPCR, was proposed more than a decade ago [28]. Another approach employing a multistep procedure of universal miRNA elongation (further designed as Uni-Elong), followed by reverse transcription and qPCR was described more recently [29]. We diluted three miRNA mimics (miR-451a, miR-375 and miR-29b) in a concentration range of 10–10^9^ molecules/reaction and quantified miRNA using three different approaches. Representative results (mimic-451a) are shown in Figure 2A. Amplification of this mimic (for instance at the starting concentrations 10^8^ molecules per reaction) was detected by Spec-TT system much earlier (Ct = 20.54) comparing to other systems (23,46–Spec-SL and 28,11 Uni-Elong). Moreover, the Uni-Elong systems reached analytic plateau at much higher concentrations of miRNA mimic, that indicated its relatively low analytic sensitivity. Comparisons of different RT-PCR systems for miR-375 and miR-29b detection gave a similar results (not shown), and the Spec-TT system was considered as optimal.

Next, we designed Spec-TT systems for other selected miRNAs and evaluated their analytic properties in a broad range of mimic concentrations (10^2^–10^13^ molecules/reaction). The area of linear dependency of amplification rate (Ct value) from the concentration of the mimic was observed in all cases, however, minimal measured mimic concentrations varied from 10^4^ to 10^7^ molecules/reaction for different Spec-TT systems. Representative results for miR-451a are shown in Figure 2B. The observed level of analytic sensitivity of the systems could become critical when analyzing the low-copied miRNAs in biological samples. To explore whether the analytic sensitivity of RT-qPCR systems is enough for measuring miRNA concentrations in thyroid tissues, twenty samples randomly selected from all groups were used to quantify ten miRNAs included in the study. Results of miR-451a quantization are shown in Figure 2B, results for other microRNAs are given in Table 1. Thus, levels of miR-451a expression in the biological samples were detected within the area of linear Ct/C (mimic-451a) relation. Among the other nine miRNAs, physiological concentrations of six molecules (miR-21, miR-29b, miR-20a, miR-146b, miR-204 and miR-375) matched the area of confident measurement by Spec-TT systems while the three other molecules (miR-126, miR-222 and miR-34a) were detectable at close-to-plateau Ct values (Table 2). Thus, the analytic properties of seven Spec-TT microRNA detection systems were deemed sufficient for further analyses, three systems were excluded.

### 2.3. Validation of Expression Alterations of Selected MiRNAs by Spec-TT RT-qPCR System

**Figure 3:**
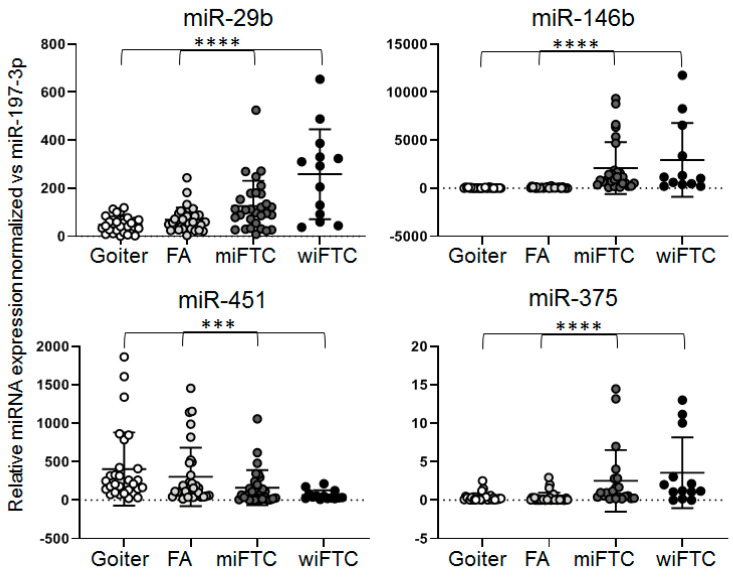
RT-qPCR analysis of selected miRNAs in individual RNA samples by Spec-TT systems.Results RT-qPCR were normalized to global mean Ct value. Statistical significance of the difference between four groups was evaluated by Kruskal–Wallis test is indicated by *** (*p* < 0.0005) and **** (*p* < 0.00005).

We then used Spec-TT RT-qPCR systems to analyze each of the selected seven miRNAs on a wider collection of pathologic thyroid tissues, including 25 FA, 30 miFTC and 20 wiFTC samples. In order to assess possible associations of miRNA expression shifts with invasive properties of thyroid epithelial cells, we included in the analysis a group of 30 goiter samples representing diffusely enlarged thyroid gland tissues. Considering the phenomena of “metastasizing FA” [2], these samples of goiter were supposed to represent “zero” level of malignant potency. Total RNA was isolated from all samples and used for RT-qPCR assay. Each of the seven miRNAs were analyzed in triplicate in every sample. The results were averaged and normalized using the global mean Ct value (mean of 735 measurements = 105 samples × 7 miRNAs). The normalized data were grouped and are presented in Table 2. Statistically significant differences between the groups were confirmed by Kruskal–Wallis test in the cases of miR-146b, -29, -375 and -451 molecules (Figure 3). Moreover, expressions of miR-146b, -29, and -375 molecules were gradually upregulated while expression of miR-451 was downregulated in a row “Goiter-FA–miFTC–wiFTC”.

### 2.4. Evaluation of Diagnostic Potency of MiRNA Pairs with Reciprocal Expression Dysregulation

Despite statistically significant differences of miRNAs expression levels, none of the miRNAs tested alone could demonstrate good predictive power in terms of differentiating between FA and FTC. This could result from the inherent variations of the miRNA expression levels and/or low amplitude of miRNAs expression alteration associated with histological diagnosis. Based on the assumption of joint regulatory activities of two or more miRNA molecules [30], we then explored diagnostic potency of amplification ratios of miRNAs with opposite (reciprocal) character of dysregulation observed in two biological statuses [24]. As shown in our previous studies, this parameter might better distinguish between two biological conditions than each of the tested miRNAs alone. Using the programmed approach, we calculated amplification ratios of all possible miRNA pairs for each sample (Ratio = 2^Ct(miR-A)–Ct(miR-B)). Obtained values were used as markers for distinguishing between groups of the samples investigated. In every case, diagnostic accuracy was evaluated by ROC analysis. Thus, two groups of FTC samples with different invasive potentials (miFTC vs. wiFTC) were hardly distinguishable with any miRNA pairs tested. In contrast, the group that consisted of both miFTC and wiFTC was well distinguishable from group of FA (Supplementary Data 2). The highest area under curve (AUC) value was obtained for the following combinations: miR-146b/miR-451a (0.92) followed by miR-21/miR-451a (0.85), miR-375/miR-451a (0.83), miR-29b/miR-451a (0.81).

**Figure 4:**
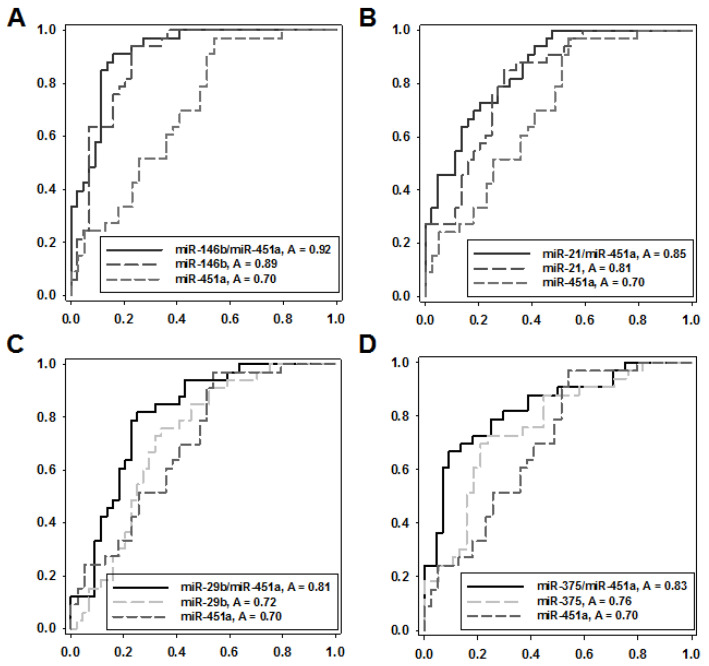
Diagnostic potency of individual miRNAs and amplification ratios of reciprocally dysregulated miRNAs pairs. ROC analysis was done with two groups of samples FA (*n*.33) and FTC (*n*.51). (**A**) miR-146b/miR-451a; (**B**) miR-21/miR-451a; (**C**) miR-29b/miR-451a; (**D**) miR-375/miR-451a.

To demonstrate improved predictive potency of reciprocally dysregulated miRNA pairs, we compared the four best miRNA pairs with this component taken alone. We used ROC analysis to estimate diagnostic potency of the miR-146b/miR-451a expression ratio, the miR-146b and the miR-451a expression levels both normalized to the global Ct mean (Figure 4A). The same calculations were performed for miR-21/miR-451a, miR-375/miR-451a and miR-29b/miR-451a pairs (Figure 4B–D). We found that the ratios of two miRNA expressions were a far more robust diagnostic criterium comparing to the analysis of the single miRNA expression in all four cases tested.

### 2.5. Systems Biology Assessment of MiRNA Pairs Opposite Dis-Regulation

**Figure 5:**
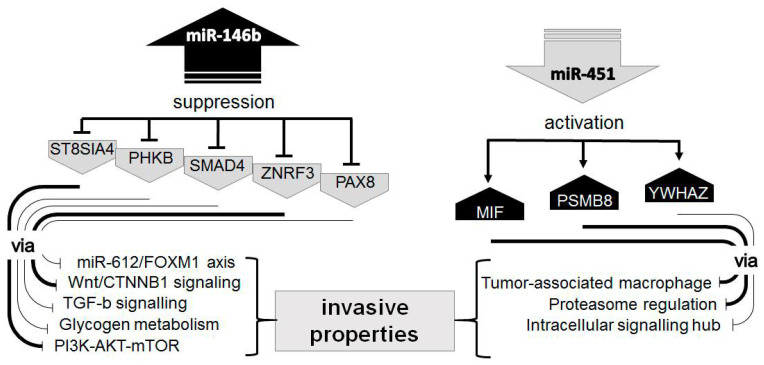
Schematic representation of miR-146b/miR-451 downstream regulatory pathways.

Obtained results implied that the phenomenon of opposite (or reciprocal) dysregulation of miRNA pairs, e.g., miR-146b and miR-451, could be associated with an increase in the invasive properties of follicular nodules and might serve as a promising diagnostic criteria. To explore whether this combined dysregulation is biologically meaningful, we used the MiRImpact method [25] that makes it possible to quantitatively assess the collective impact of altered regulations of multiple microRNAs on the activities of intracellular molecular pathways. To this end, microRNA-pathway activation strength (miPAS) values were calculated that reflected the influence of microRNA expression levels on the activation of multiple molecular pathways. We separately performed miPAS analyses for all the microRNA pairs under investigation. As the input data, we used expression change (fold change) of each of microRNA for every pair that has been observed experimentally in the groups of FA and FTC samples (Table 3). In this way, we identified several tumor invasion-related pathways that had been modelled to be affected by the apparent changes of microRNA concentrations. Calculated values of miRAS are presented in Table 3. This table also includes biomarker robustness characteristics (AUC, sensitivity, specificity) for each oppositely regulated pair of microRNAs. There was also a coincidence detected between the diagnostic robustness of miRNA pairs and their predicted abilities to activate invasion/metastasis related signaling pathways. Thus, the miR-146b/miR-451a pair had a maximum diagnostic accuracy and lead to the most significant modelled activation of cell adhesion and migration-promoting pathways, i.e., *PTEN*/Pathway Adhesion or Migration, *PTEN*/Pathway Migration, KEGG/Gap junction main pathway, KEGG/Focal adhesion main pathway, KEGG/Regulation of actin cytoskeleton main pathway. Moreover, reciprocal dysregulation of miR-146b/miR-451 could result in considerable dysregulation of many invasion-related genes (Figure 5) Other combinations of reciprocally regulated miRNAs had lower diagnostic values and showed smaller impact on the regulation of tumor invasion pathways. The predicted impacts of particular miRNA pairs on invasion/metastasis–related molecular pathways are schematically shown in Supplementary Data 3.

## 3. Discussion

Development of new miRNA-based methods for differential diagnostics of thyroid nodules is an active research area. A great number of studies were performed with different designs and scales while with the common purpose to identify miRNA signatures of the specific types of thyroid tumors. Interesting results were achieved by integrated characterization of the genetic, epigenetic, gene and miRNA expression alterations in thyroid carcinomas [31]. The different miRNA profiling technologies were applied with a goal to identify miRNA differentially expressed in FTC and FA: llumina platform [32], IPMC custom microarray [18] and Exiqon platform [33] were used. The various analytic approaches were explored to optimize analysis of the global expression data sets. Stakowsky et al. combined data from multiple studies (totally 59 FTC and 44 FA samples) and performed cross-comparison evaluation of FTC-specific miRNA signatures [20]. After estimating differentially expressed protein-coding genes in 25 FTC and 25 FA samples, Hossain et al. went ahead to identify potentially important pathways and implicated miRNAs using Gene Ontology (GO) and the Kyoto Encyclopedia of Genes and Genomes (KEGG) resources [34]. The first approach revealed 13 differentially expressed miRNAs (hsa-miR-7-5p, -7-2-3p, -486-5p, -144-5p, -30a-3p, -let-7d-5p, -135a-5p, -612, -637, -21-3p, -874-3p, -571, and -222-3p) while second one came out with 10 molecules (hsa-mir-335-5p, -26b-5p, -124-3p, -16-5p, -192-5p, -1-3p, -17-5p, -92a-3p, -215-5p, and -20a-5p). There was no overlap observed. This discrepancy may indicate a gap between detectable differences in FTC vs. FA miRNA expression patterns and current understanding of miRNA involvement in cellular regulatory pathways. Despite a huge amount of work in the field being done, miRNA-based approaches for differential diagnostic of follicular thyroid node are still far from the desired level of performance. In planning our research, we intended to consider the two most problematic, in our opinion, issues hampering development and clinical implementation of miRNA-based diagnostic tests.

First, if we do not exactly know the key molecular mechanisms mediating malignant phenotypes of follicular thyroid cells, we should consider clinically important and histologically distinguishable features of tumors. Since invasive behavior is a main property of FTC distinguishing it from FA, we composed groups of samples of follicular thyroid nodules with different invasive potencies (FA, minimally invasive FTC and widely invasive FTC) and were looking for miRNAs with associated expression alterations. We supposed, that identified molecules (38 from 170 profiled miRNAs) have a high probability to be involved in the post-transcriptional regulation of invasion-related genes. Despite a relatively low number of biological samples included and miRNAs tested, our approach allowed us to focus further research on the most relevant molecules.

Second, in routine clinical practice, miRNA quantification will be most probably performed by RT-qPCR, therefore we attempted to optimize this technology. Despite broad application, RT-qPCR analysis of miRNA is still not a trivial issue due to (i) the small size of molecules and (ii) the absence of a confident method of data normalization. In our study, we a did direct comparison of several approaches to elongate and reverse transcribe miRNA and selected Spec-TT RT-qPCR technology, as proposed by P. Androvic [27]. This method, with slight modifications, revealed the best analytic sensitivity compared to other methods [28,29]. However, even the best analytic system did not have enough sensitivity to confidently quantify three from 10 miRNAs due to either individual analytic properties of Spec-TT RT-qPCR systems or low concentration of the molecules analyzed. Thus, it seems to be important to confirm sufficient analytic properties of methods with regard to each particular miRNA. As far as we know, this issue was not being addressed in any previous studies and might present one of the possible causes for the results discrepancies. Another problematic issue is the normalization of RT-qPCR results. With a small number of analyzed miRNAs, the method of normalization may have considerable impact on the results. Various non-coding RNAs (SNORD95 RNU6A, RNU6B) or miRNAs (hsa-miR-151a-3p, -197-3p, -99a-5p and-214-3p) were proposed as “endogenous references” for analysis of miRNAs in thyroid tissue [20,26,35], indicating an absence of confident reference. To resolve this problem, we proposed a method based on the calculation of ratios of Ct values for two miRNAs with reciprocal character of invasion-associated expression alteration. This parameter reflects expression changes of two disease-relevant miRNAs and does not require normalization. We have explored this approach in some previous studies [24,36] and shown a high diagnostic value of proposed parameters comparing to single miRNAs. Moreover, we noted that optimal diagnostic miRNA pairs are frequently not composed by “top UP” and “top DOWN”—regulated molecules and might be formed by miRNAs with intermediate range of dysregulation. This observation can reflect phenomena of biologically linked reciprocal dysregulation of two miRNAs that, however, need to be confirmed and deeper investigated. In the presented study, we made a first attempt to find out whether the diagnostically significant reciprocal dysregulation of two miRNAs is not just a random event but may have biological relevance. Using the MiRImpact method, we modelled the functional impacts of microRNA pairs dysregulation on the activities of molecular pathways. For the pair of miR-146b/miR-451 we simulated activation of the biggest number of tumor invasion-/metastasis- regulating signaling pathways compared to the other miRNAs pairs.

The over-expression of miR-146b was described in different types of thyroid cancers, including follicular carcinomas, by many authors. Moreover, over-expression of miR-146b in immortalized thyroid epithelial cells HTori and metastatic follicular thyroid carcinoma cells FTC-133 significantly induced cells migration [37]. Molecular evens downstream of over-expressed miR-146b were also studied in the context of thyroid cancer [38]. A list of the direct targets of miR-146b includes several genes involved in the maintenance of malignant phenotypes and invasive properties (Figure 5): *PHKB* [39,40], *SMAD4* [41,42], *PAX8* [43], *ZNRF3* [44] and *ST8SIA4* [45]. The last two miR-146b downstream pathways are shown to be involved in thyroid carcinogenesis in independent studies. The cancer-associated suppression of miR-451 is also well studied, including in relation to thyroid cancer [46]. Some of miR-451 downstream targets are well known to be involved in the control of invasive properties of thyroid and others cancers (Figure 5): *MIF* [46,47] and *PSMB8* [48,49]. The regulatory link miR-451-*YWHA2* is shown to be essential during embryogenesis and observed in breast and hepatocellular cancers [50,51]. The oncogenic role of *YWHA2* was investigated in breast, colon, hepatocellular, lung, gastric and prostate cancers and summarized recently [52]. Interesting to note, is that *YWHA2* was once evaluated as one of the six best reference genes in thyroid specimens [53], that might indicate a robust expression control and important function of this protein. Therefore, miR-451-mediated dysregulation of *YWHA2* might have a great impact on thyroid cells biology.

Thus, the results of our investigation are supported by multiple independent studies. The miR-146b/miR-451 pair dysregulation appeared to be an important event associated with malignant transformation and invasive properties of follicular thyroid nodes. We plan to expand the analytic approach presented in this report to identify additional reciprocally dysregulated miRNAs and to develop a robust method for differential diagnostics of FA and FTC.

## 4. Materials and Methods

### 4.1. Patients and Samples

The present study was approved by the local Ethics Committee of the Federal State Budgetary Institution “Scientific Medical Research Center of Oncology named after N.N. Petrov of the Ministry of Health of Russia” as of 29 October 2018 (Internal No. 2/173). Patient participations was voluntary and required informed consent. Clinical characteristics of the patients are presented on Table 4. Before being included into analyses, the clinical material and information were subjected to depersonalization. Tissue samples were processed according to the standard protocol. After macroscopic evaluation, thyroid nodules were fixed in 10% neutral buffered formalin, dehydrated in a graded series of alcohols, and embedded in paraffin. The 4–5 μm thick paraffin sections were stained with hematoxylin and eosin. After histological evaluation by two pathologists independently, 8–10 sections (10 μm thickness) from each sample were used for RNA isolation.

### 4.2. RNA Isolation

Isolation of RNA from histological preparations was performed according to the protocol: 1 mL of mineral oil (MP Biomedicals, Irvine, CA, USA) was added to the tube with tissue sections, mixed in a vortex and incubated in a thermal shaker at 65 °C for 2 min. After centrifugation for 5 min at 16,000× *g*, the supernatant was completely discarded, and the pellet was consequently washed with 96% and 70% ethanol and dried. Next, 700 μL of guanidine lysis buffer (4 M guanidine isothiocyanate, 25 mM sodium citrate, 0.3% sarkosyl, 3% DTT) was added to the tube. The sample was mixed and left in a thermal shaker for 15 min at 65 °C. After centrifugation at 10,000× *g* for 2 min, the supernatant was transferred into new tubes, followed by the addition of 600 μL of isopropanol and RNA absorbing paramagnetic particles (Sileks Ltd., Moscow, Russia), mixing and incubation at room temperature for 5 min. Magnetic-separation rack was used to separate the magnetic particles, that were washed consequently with 70% ethanol and acetone, and dried. Finally, the RNA was eluted in 100 μL RNAse-free water. If not analyzed immediately, RNA samples were stored at −20 °C until further use. The concentration and quality of RNA were evaluated on a NanoPhotometer Implen N50 (Implen GmbH, Munchen, Germany).

### 4.3. RT-QPCR

In order to perform preliminary miRNA profiling three RNA pools presenting FA (*n*.10), miFTC (*n*.10) and wiFTC (*n*.10) were prepared. The RNA was first polyadenylated, then reverse transcribed using miRCURY LNA Universal RT microRNA Polyadenylation and cDNA synthesis Kit (Qiagen, Germantown, MD, USA). The quantitative PCR assay of 170 miRNAs was performed using miRCURY LNA miRNA PCR Panel (kat. N. 339325; Qiagen, Germantown, MD, USA). In order to see if results of pooled samples analysis represented well groups of samples, two miRNAs (miRNA-126 and miRNA-146b) were tested in each of 30 samples using individual assays.

In order to select the best approach for individual miRNA analysis, we have tested three alternative technologies of RT-qPCR. Two of them, designed as spec-SL [28] and spec-TT [22], supposed miRNA-specific reverse transcription. Another method, designed as Uni-elong [29], included consequent steps of miRNA elongation and reverse transcription with universal RT primer. Sequences of all RT primers, adaptors, PCR primers and probe (presented in Supplementary Data 4) were purchased from Algimed-Techno Ltd. (Minsk, Belarus) as well as synthetic mimics of miRNAs. The enzymes used in our study included T4 RNA Ligase 2/truncated K227Q (New England Biolabs GmbH, Frankfurt am Main, Germany), PrimeScriptTM 1st strand cDNA Synthesis Kit (TaKaRa Bio Inc, Mountain View, CA, USA), M-MuLV-RH kit and BioMaster HS-qPCR kit (both from BioLabMix Ltd., Novosibirsk, Russia).

After optimal technology (Spec-TT) was selected, analytic systems for 10 miRNAs were validated with a broad range of mimics concentrations. Validated systems were used for analysis of all samples included in the study in according to following protocol. Reverse transcription (RT) assays were performed using 1 μL RNA and 0.2 μM of TT-RT primes with M-MuLV-RH RT kit in 20 μL reaction volume. The reaction was carried out at 25 °C for 45 min, followed by incubation at 85 °C for 5 min to inactivate reverse transcriptase. Quantitative PCR was performed using 2 μL RT reaction mix, PCR primers 1.2 μM both and FAM-labeled zond 0.8 μM with BioMaster HS-qPCR kit. Following conditions for qPCR were used: 95 °C for 10 min and 45 cycles of 95 °C for 5 s followed by 65 °C for 15 s. RT-qPCR reactions for each miRNA molecule were repeated independently twice and averaged.

All reactions were performed with CFX96 Touch™ Real-Time PCR Detection System (Bio-Rad Laboratories, Pleasanton, CA, USA). Obtained data were analyzed with CFX Manager 3.1 (Bio-Rad Laboratories, Pleasanton, CA, USA), SigmaPlot 11.0 (Systat Software, Inc., San Jose, CA, USA) and GraphPad Prism 8.0.2 (GraphPad Software, San Diego, CA, USA) Statistical significance of observed difference between grouped results was estimated using non-parametric Mann–Whitney test in case of two groups comparison and Kruskal–Wallis test in case of multiple groups comparison.

### 4.4. Estimation of Reciprocally Dysregulated MiRNAs Pair Diagnostic Value

To explore diagnostic utility of paired miRNAs combinations, an exhaustive search of pairs of dysregulated miRNAs with best diagnostic potency was performed using ROC analysis. This was done programmatically by forming reciprocal pairs as all possible combinations of selected set of miRNAs, calculating amplification ratios (Ratio = $2^{Ct\left(miR-A\right)–Ct\left(miR-B\right)}$). Since these values were calculated for each sample, they were used as a discriminative marker for distinguishing between groups of samples and estimating diagnostic accuracy. Total number of analyzed pairs could be estimated as $P_{n}^{r}\;=\;\frac{n!}{\left(n-r\right)!}$ (a number of ordered samples of size R, without replacement, from N objects). Results of exhaustive search were sorted by diagnostic value and analyzed manually by plotting the ROC-curve for the most notable ones.

### 4.5. Molecular Pathways Evaluation and Pathway Activation Analysis

We used the Oncobox signalling pathways knowledge base [54,55] to determine the structures of intracellular pathways and relative functions of their individual members. For the annotation of miRNA dysregulation effects on molecular pathway activation levels, we applied the MiRImpact method [25]. It utilizes microRNA expression data, attached to a specific miR target database, and calculates miR pathway activation strength (miPAS). The formula for miPAS calculation operates with miR expression data and the knowledge on the mRNA targets for each microRNA under investigation. For a certain pathway *p*, miPASp = ∑*n* (−ARRn, *p*) ∑k log (miCNRn, k).

Here summation is performed for all gene products participating in a pathway *p*. The role of every gene product in a pathway *p* is reflected by a Boolean activator/repressor role (ARRnp) parameter, which is set equal to 1 for a pathway activator gene product, −1 for a repressor, and intermediate values −0.5, 0.5, and 0 for the gene products with repressor, activator, or unknown roles, respectively. Furthermore, inner summation is done over all microRNAs affecting pathway *p*. The miCNRk value (microRNA case-to-normal ratio) is the ratio of the expression level of microRNAk in the biosample under study to its average expression level in the control samples. The positive value of miPASp indicates upregulation of a pathway *p*, and the negative value means its repression by overall profile of microRNAs observed in a sample under investigation.

### 4.6. MicroRNA Target Databases and MiRImpact Settings

We collected, analyzed, and preprocessed data from experimentally validated microRNA target databases miRTarBase [56] and Diana TarBase [57] according to [58] to add microRNA–mRNA interaction data to the MiRImpact operational database. For miPAS calculations, we treated microRNA concentrations in FTC as the “case” and in FA as the “normal” values. The results of differential pathway activation analysis were visualized using the Oncobox pathway visualization tool [59].

## Figures and Tables

**Table 1:** Analytic properties of spec-TT RT-qPCR system for miRNA detection.

	Synthetic MiRNA		Biological RNA Samples
miRNA	Plateau (Ct)	Minimal MiRNA Concentration Detected (Molecules/Reaction)	Interval (Ct)
miR-21	From 31.8	10^7^	From 20.3 to 31.1
miR-29b	From 32.8	10^4^	From 20.3 to 24.9
miR-30a	From 28.5	10^6^	From 18.9 to 24.4
miR-146b	From 24.9	10^7^	From 20.3 to 24.6
miR-375	From 30.1	10^7^	From 25.9 to 39.8
miR-451	From 33.4	10^6^	From 17.9 to 23.8

**Table 2:** Relative miRNA expression in various types of thyroid nodes.

	MiR-29b	MiR-375	MiR-451	MiR-146b	MiR-21	MiR-30a	MiR-204
Goiter	81.51	0.34	403.78	70.33	165.76	171.04	0.16
*SEM*	*24.70*	*0.10*	*88.31*	*23.12*	*27.85*	*42.97*	*0.04*
FA	69.24	0.30	30.85	75.07	62.88	175.93	1.42
*SEM*	*8.67*	*0.11*	*66.15*	*10.54*	*11.19*	*24.54*	*0.44*
miFTC	125.66	5.17	161.95	1960.68	263.69	495.07	119.09
*SEM*	*19.20*	*2.23*	*41.63*	*484.85*	*33.90*	*84.44*	*112.97*
wiFTC	239.80	0.44	65.09	1344.30	114.20	635.06	60.67
*SEM*	*51.42*	*0.29*	*16.63*	*835.22*	*18.14*	*362.06*	*44.70*

**Table 3:** MicroRNA-pathway activation strength (miPAS) values reflecting the impacts of microRNA concentrations on the activation of molecular pathways.

		MiR146b/MiR-451a	MiR375/MiR-451a	MiR21/MiR-451a	MiR30a/MiR-451a	miR29b/MiR-451a
SpecTT-RT-qPCR	Experimentally observed miRNA expression alteration (fold change between FA and FTC groups)	22.01/0.37	9.35/0.37	3.00/0.37	3.21/0.37	2.64/0.37
MiRImpact method	PTEN Pathway Adhesion or Migration	3.16	2.44	-	1.12	-
	PTEN Pathway Migration	3.16	-	-	-	-
	KEGG Gap junction Main Pathway	3.16	1.60	1.23	-	-
	KEGG Focal adhesion Main Pathway	2.32	-	-	-	-
	KEGG Adherens junction Main Pathway		2.44	1.23	-	-
	KEGG Regulation of actin cytoskeleton Main Pathway	3.16	-	1.23	1.12	-
ROC analysis	Diagnostic robustness, FA vs. FTC discrimination (AUC)	0.91	0.88	0.88	0.73	0.7
	Sensitivity (%)	84.85	78.79	72.33	78.79	81.82
	Specificity (%)	86.36	75	79.55	75	75

**Table 4:** Patients included in the studies.

	Gender (M/F)	Age Averaged	Number
Goiter	3/27	63/51	30
Follicular adenoma (FA)	5/28	52/54	33
FTC minimally invasive (miFTC)	4/30	44/47	34
FTC widely invasive (wiFTC)	6/11	64/48	17

## Supplementary Materials

The following are available online at https://www.mdpi.com/1422-0067/21/17/5950/s1. S1: Pooled samples (FA, *n*.10; miFTC *n*.10; wiFTC *n*.10) miRNA profiling, S2: Results of ROC AUC calculation for all possible miRNA pairs, S3: Predicted impacts of particular miRNA pairs on invasion/metastasis–related molecular pathways (schemas), S4: Sequences of primers and probes.

## Abbreviations

FTC	Follicular thyroid cancer
miFTC	Minimally invasive follicular thyroid cancer
wiFTC	Widely invasive follicular thyroid cancer
FA	Follicular adenoma
Spec-TT	Reverse Transcription system with miRNA-specific two-tailed primer
Spec-SL	Reverse Transcription system with miRNA-specific spem-looped primer
Uni-Elong	Reverse Transcription system with elongation and universal primer
