# Supplementary material for: Reciprocal Dysregulation of MiR-146b and MiR-451 Contributes in Malignant Phenotype of Follicular Thyroid Tumor
Source: Int J Mol Sci. 2020 Aug 19;21(17):5950. doi: 10.3390/ijms21175950 (PMC7503510; doi:10.3390/ijms21175950)
Supplement: Supplementary file 1 [file ijms-21-05950-s001.zip › Suppl 1. Exiqon_miR-Expression profiling data.pdf]

Knyazeva et all., Reciprocal dysregulation of mir-146b and mir-451 contributes in malignant phenotype of follicular thyroid tumor.

Supplementary data 2. MiRNA expression profiling of FA, miFTC and wiFTC using pooled samples

*MiRNA selected for further analysis are marked

**miRNA**	**normalized to global Ct mean**			**Log(2)**		
	**FA**	**miFTC**	**wiFTC**	**FA**	**miFTC**	**wiFTC**
hsa-let-7a-5p	7,765	7,631	15,102	2,957	2,932	3,917
hsa-let-7b-3p	0,057	0,052	0,062	-4,130	-4,257	-4,008
hsa-let-7b-5p	1,426	1,915	3,600	0,512	0,938	1,848
hsa-let-7d-3p	0,332	0,375	0,838	-1,589	-1,414	-0,254
hsa-let-7d-5p	0,521	0,581	0,928	-0,939	-0,785	-0,107
hsa-let-7f-5p	2,612	1,808	2,056	1,385	0,854	1,040
hsa-let-7g-5p	3,176	3,898	5,096	1,667	1,963	2,349
hsa-let-7i-5p	1,774	2,780	2,593	0,827	1,475	1,375
hsa-miR-101-3p	1,209	1,310	0,550	0,273	0,390	-0,862
hsa-miR-106b-5p	0,410	0,511	0,266	-1,286	-0,968	-1,913
hsa-miR-10b-5p	0,071	0,134	0,080	-3,811	-2,904	-3,641
**hsa-miR-126-5p**	**9,934**	**8,516**	**2,180**	**3,312**	**3,090**	**1,124**
hsa-miR-125b-5p	0,259	0,417	0,650	-1,947	-1,261	-0,622
hsa-miR-126-3p	0,303	0,459	0,510	-1,723	-1,123	-0,971
hsa-miR-128-3p	0,089	0,086	0,096	-3,486	-3,534	-3,383
hsa-miR-130a-3p	0,223	0,304	0,303	-2,162	-1,719	-1,722
hsa-miR-130b-3p	0,015	0,013	0,022	-6,100	-6,210	-5,503
hsa-miR-132-3p	0,084	0,002	0,057	-3,569	-8,818	-4,138
hsa-miR-133a-3p	0,100	2,006	0,055	-3,327	1,005	-4,175
hsa-miR-133b	0,105	1,232	0,039	-3,247	0,301	-4,686
hsa-miR-139-5p	0,127	0,076	0,132	-2,979	-3,717	-2,918
**hsa-miR-21-3p**	**0,259**	**0,417**	**0,650**	**-1,947**	**-1,261**	**-0,622**
**hsa-miR-204-5p**	**0,303**	**0,459**	**0,510**	**-1,723**	**-1,123**	**-0,971**
hsa-miR-140-3p	0,193	0,233	0,244	-2,374	-2,103	-2,036
hsa-miR-142-3p	1,823	3,047	0,843	0,866	1,608	-0,246
hsa-miR-142-5p	0,086	0,204	0,064	-3,543	-2,292	-3,957
hsa-miR-143-3p	3,726	1,837	3,466	1,898	0,877	1,793
hsa-miR-144-3p	0,284	0,437	0,104	-1,817	-1,194	-3,270
hsa-miR-144-5p	0,061	0,039	0,017	-4,031	-4,685	-5,908
hsa-miR-145-5p	4,034	4,605	2,217	2,012	2,203	1,149
**hsa-miR-146b-5p**	**0,746**	**1,478**	**2,741**	**-0,423**	**0,564**	**1,455**
hsa-miR-148a-3p	2,088	0,893	1,135	1,062	-0,163	0,182
hsa-miR-148b-3p	0,807	0,739	1,714	-0,309	-0,436	0,777
hsa-miR-150-5p	0,612	0,626	0,558	-0,708	-0,675	-0,842
hsa-miR-151a-5p	0,762	1,116	1,064	-0,392	0,159	0,090

hsa-miR-152-3p	0,480	0,358	0,432	-1,059	-1,483	-1,212
hsa-miR-154-5p	0,003	0,006	0,002	-8,373	-7,314	-8,887
hsa-miR-15b-3p	0,023	0,025	0,028	-5,450	-5,315	-5,145
hsa-miR-15b-5p	0,087	0,148	0,007	-3,531	-2,754	-7,131
hsa-miR-16-2-3p	0,009	0,024	0,016	-6,794	-5,352	-5,952
hsa-miR-16-5p	6,224	9,004	5,397	2,638	3,171	2,432
hsa-miR-17-5p	0,005	0,005	0,002	-7,755	-7,573	-8,968
hsa-miR-186-5p	0,021	0,030	0,024	-5,588	-5,052	-5,356
hsa-miR-18a-5p	0,028	0,029	0,018	-5,176	-5,103	-5,814
hsa-miR-18b-5p	0,014	0,028	0,011	-6,134	-5,144	-6,544
hsa-miR-193a-5p	0,017	0,019	0,053	-5,921	-5,689	-4,238
hsa-miR-195-5p	0,244	0,293	0,155	-2,037	-1,773	-2,693
hsa-miR-197-3p	0,087	0,132	0,195	-3,527	-2,917	-2,361
hsa-miR-199a-3p	0,447	0,730	0,482	-1,161	-0,455	-1,052
hsa-miR-199a-5p	0,406	0,702	0,809	-1,300	-0,511	-0,306
hsa-miR-19a-3p	1,033	2,093	0,504	0,047	1,066	-0,990
hsa-miR-19b-3p	2,711	4,757	1,425	1,439	2,250	0,511
hsa-miR-200a-3p	0,318	0,480	0,273	-1,651	-1,059	-1,871
hsa-miR-20a-5p	0,471	0,832	0,338	-1,087	-0,265	-1,563
hsa-miR-2110	0,012	0,011	0,008	-6,354	-6,483	-6,888
hsa-miR-215-5p	0,047	0,041	0,051	-4,423	-4,612	-4,299
hsa-miR-21-5p	0,067	11,008	4,889	-3,900	3,460	2,290
hsa-miR-221-3p	0,560	1,193	0,551	-0,838	0,254	-0,861
**hsa-miR-222-3p**	**0,460**	**0,545**	**1,262**	**-1,121**	**-0,875**	**0,335**
hsa-miR-22-3p	0,721	0,579	0,755	-0,471	-0,789	-0,406
hsa-miR-22-5p	0,088	0,104	0,061	-3,499	-3,261	-4,046
hsa-miR-23a-3p	4,108	5,409	4,739	2,039	2,435	2,245
hsa-miR-23b-3p	4,081	5,144	4,295	2,029	2,363	2,103
hsa-miR-25-3p	0,425	0,462	0,422	-1,236	-1,114	-1,244
hsa-miR-26b-5p	0,580	0,485	0,510	-0,787	-1,044	-0,972
hsa-miR-27b-3p	3,144	2,633	4,067	1,652	1,397	2,024
hsa-miR-28-5p	0,107	0,002	0,095	-3,226	-8,660	-3,402
hsa-miR-29a-3p	3,526	4,545	4,694	-0,413	1,767	2,231
**hsa-miR-29b-3p**	**1,368**	**3,353**	**4,634**	**0,452**	**1,746**	**2,212**
hsa-miR-301a-3p	0,313	0,460	0,484	-1,675	-1,121	-1,047
hsa-miR-30c-5p	6,616	7,609	5,034	2,726	2,928	2,332
**hsa-miR-30a-5p**	**0,712**	**0,625**	**0,344**	**-0,490**	**-0,678**	**-1,539**
hsa-miR-30e-3p	0,200	0,263	0,255	-2,321	-1,927	-1,970
hsa-miR-30e-5p	0,911	1,323	0,625	-0,134	0,404	-0,677
hsa-miR-320a	0,258	0,260	1,040	-1,954	-1,941	0,056
hsa-miR-320b	0,071	0,034	0,177	-3,820	-4,867	-2,496
hsa-miR-320c	0,030	0,032	0,132	-5,062	-4,973	-2,918
**hsa-miR-34a-5p**	**0,343**	**1,074**	**1,089**	**-1,544**	**0,103**	**0,123**
**hsa-miR-375-3p**	**1,089**	**1,461**	**2,332**	**0,123**	**0,547**	**1,221**
hsa-miR-324-3p	0,154	0,124	0,259	-2,699	-3,013	-1,948
hsa-miR-328-3p	0,466	0,407	0,687	-1,101	-1,296	-0,542
hsa-miR-335-5p	0,007	0,012	0,007	-7,235	-6,394	-7,097

hsa-miR-338-3p	0,185	0,274	0,140	-2,433	-1,867	-2,832
hsa-miR-339-3p	0,032	0,021	0,056	-4,986	-5,567	-4,147
hsa-miR-342-3p	0,230	0,201	0,210	-2,122	-2,313	-2,249
hsa-miR-362-3p	0,106	0,168	0,026	-3,244	-2,576	-5,246
hsa-miR-365a-3p	0,253	0,326	0,229	-1,984	-1,615	-2,129
hsa-miR-374a-5p	0,059	0,189	0,036	-4,072	-2,401	-4,816
hsa-miR-374b-5p	0,169	0,156	0,075	-2,567	-2,678	-3,730
hsa-miR-376a-3p	0,004	0,010	0,003	-7,996	-6,586	-8,603
hsa-miR-423-3p	0,593	0,678	0,881	-0,755	-0,560	-0,183
hsa-miR-423-5p	0,137	0,198	0,422	-2,870	-2,338	-1,244
hsa-miR-424-5p	1,072	0,222	0,908	0,101	-2,174	-0,139
hsa-miR-425-5p	0,126	0,134	0,113	-2,983	-2,899	-3,145
**hsa-miR-451a-5p**	**3,812**	**1,439**	**0,823**	**1,931**	**0,525**	**-0,281**
hsa-miR-486-5p	0,038	0,034	0,035	-4,736	-4,873	-4,856
hsa-miR-497-5p	0,389	1,479	0,907	-1,363	0,565	-0,140
hsa-miR-501-3p	0,022	0,007	0,027	-5,529	-7,150	-5,220
hsa-miR-574-3p	0,180	0,117	0,215	-2,477	-3,094	-2,215
hsa-miR-590-5p	0,030	0,032	0,016	-5,043	-4,947	-5,956
hsa-miR-629-5p	0,008	0,006	0,006	-7,016	-7,357	-7,426
hsa-miR-652-3p	0,124	0,146	0,228	-3,012	-2,776	-2,133
hsa-miR-660-5p	0,175	0,127	0,098	-2,512	-2,974	-3,355
hsa-miR-7-1-3p	0,019	0,013	0,013	-5,737	-6,242	-6,221
hsa-miR-7-5p	0,130	0,155	1,084	-2,940	-2,693	0,117
hsa-miR-766-3p	0,005	0,007	0,018	-7,538	-7,073	-5,758
hsa-miR-874-3p	0,144	0,329	0,439	-2,798	-1,605	-1,189
hsa-miR-877-5p	0,007	0,002	0,023	-7,235	-8,671	-5,440
hsa-miR-93-5p	0,374	0,439	0,567	-1,420	-1,187	-0,819
hsa-miR-99b-5p	0,465	0,501	0,983	-1,105	-0,997	-0,025
