# Supplementary material for: Reciprocal Dysregulation of MiR-146b and MiR-451 Contributes in Malignant Phenotype of Follicular Thyroid Tumor
Source: Int J Mol Sci. 2020 Aug 19;21(17):5950. doi: 10.3390/ijms21175950 (PMC7503510; doi:10.3390/ijms21175950)
Supplement: Supplementary file 1 [file ijms-21-05950-s001.zip › Suppl 2. ROC analysis results (AUC).pdf]

Knyazeva et all., Reciprocal dysregulation of mir-146b and mir-451 contributes in malignantphenotype of follicular thyroid tumor.

Supplementary data 3. AUC value possible pairs of RNA

	miRNA pairs	AUC
1	hsa-miR-146-5p/hsa-miR-451a	0.915
2	hsa-miR-375-3p/hsa-miR-451a	0.862
3	hsa-miR-21-5p/hsa-miR-451a	0.860
4	hsa-miR-30-5p/hsa-miR-451a	0.820
5	hsa-miR-29b-3p/hsa-miR-451a	0.817
6	hsa-miR-146b-5p/hsa-miR-30-5p	0.809
7	hsa-miR-375-3p/hsa-miR-30-5p	0.767
8	hsa-miR-375-3p/hsa-miR-29b-3p	0.735
9	hsa-miR-146b-5p/hsa-miR-29b-3p	0.730
10	hsa-miR-146b-5p/hsa-miR-204-5p	0.695
11	hsa-miR-375-3p /hsa-miR-204-5p	0.680
12	hsa-miR-21-5p /hsa-miR-204-5p	0.675
13	hsa-miR-204-5p/hsa-miR-451a	0.663
14	hsa-miR-375-3p /hsa-miR-21-5p	0.631
15	hsa-miR-21-5p /hsa-miR-29b-3p	0.625
16	hsa-miR-146b-5p/hsa-miR-21-5p	0.604
17	hsa-miR-29b-3p/hsa-miR-204-5p	0.601
18	hsa-miR-146b-5p/hsa-miR-375-3p	0.581
19	hsa-miR-21-5p /hsa-miR-30-5p	0.572
20	hsa-miR-375-3p /hsa-miR-146b-5p	0.556
21	hsa-miR-30-5p/hsa-miR-204-5p	0.544
22	hsa-miR-29b-3p/hsa-miR-30-5p	0.519
23	hsa-miR-21-5p /hsa-miR-375-3p	0.505
