# Supplementary material for: Reciprocal Dysregulation of MiR-146b and MiR-451 Contributes in Malignant Phenotype of Follicular Thyroid Tumor
Source: Int J Mol Sci. 2020 Aug 19;21(17):5950. doi: 10.3390/ijms21175950 (PMC7503510; doi:10.3390/ijms21175950)
Supplement: Supplementary file 1 [file ijms-21-05950-s001.zip › Suppl 4. Primers and probes.pdf]

Knyazeva et all., Reciprocal dysregulation of mir-146b and mir-451 contributes in malignant phenotype of follicular thyroid tumor.

Supplementary data 1. Primers and probes used for RT-qPCR

miR-29b MI0000105	Synthetic miRNA		UAGCACCAUUUGAAAUCAGUGUU
	Spec-TT	Primer RT	CAAATGGTCGACGAATACTGCTAGAGTTGC TAGCAGAGCCCTTAAAACACT
		PCR-forward	CAAATGGTCGACGAATACTG
		PCR-reverse	GGTAGCACCATTTGAAATCAG
		Probe	AGAGTTGCTAGCAGAGCCCTTAA
	Spec-SL	Primer RT	GTCGTGTCTGAGGCTCACTGAGACCTTTCGAC CCTCGACACGACAA(C)AC(T)GAT
		PCR-forward	CAGCACTAGCACCATTTGAA
		PCR-reverse	CTGAGGCTCACTGAGACCT
		Probe	TTCGCACCCTCGACAGACAACACTGAT
	Uni-Elong	Adaptor	GGCCGAACTACGACCTGCATAAACGG
		Primer RT	CCCAGTTATGGCCGTTTATGCAGGT
		PCR-forward	TAGCACCATTTGAAATCAGTGTT
		PCR-reverse	CCCAGTTATGGCCGTTTA
miR-375 MI0000783	Synthetic miRNA		UUUGUUCGUUCGGCUCGCGUGA
	Spec-TT	Primer RT	CGAACAAATCAAGCTCTCCAGGTACAGTTGGTACCTGACTCCACG CTCACGC
		PCR-forward	CGAACAAATCAAGCTCTCCAG
		PCR-reverse	CGGTTTGTTCGTTCGGCTC
		Probe	ACAGTTGGTACCTGACTCCACGC
	Spec-SL	Primer RT	GTCGTGTCAGAGGCTCACTGAGACCTATTCGC ACCTCGACACGACT(C)ACG(C)GA

		PCR-forward	ACAGCTTTGTTCGTTCGGC
		PCR-reverse	CTGAGGCTCACTGAGACCT
		Probe	CGACACGACTCACGCGA
	Uni-Elong	Adaptor	GGCCGAACTACGACCTGCATAAACGG
		Primer RT	CCCAGTTATGGCCGTTTATGCAGGT
		PCR-forward	TTGTTCGTTCGGCTCGC
		PCR-reverse	CCCAGTTATGGCCGTTTA
mir451a-5p MI0001729	Synthetic miRNA		AAACCGUUACCAUUACUGAGUU
	Spec-TT	Primer RT	AACGGTTTCGACGAATACTGCTAGAGTTGCTAGCAGAGCCCTTAAAACTCA
		PCR-forward	CAACGGTTTCGACGAATAC
		PCR-reverse	GGAAACCGTTACCATTACTG
		Probe	AGAGTTGCTAGCAGAGCCCTTAA
	Spec-SL	Primer RT	GTCGTGCTGAGGCTCACTGAGACCTATTCGCACCTCGACACGACAA(C)T(C)AGT
		PCR-forward	CCAGCAAACCGTTACCATT
		PCR-reverse	CTGAGGCTCACTGAGACCT
		Probe	CGCACCTCGACACGACAACTCAGTA
	Uni-Elong	Adaptor	GGCCGAACTACGACCTGCATAAACGG
		Primer RT	CCCAGTTATGGCCGTTTATGCAGGT
		PCR-forward	AAACCGTTACCATTACTGAGTT
		PCR-reverse	CCCAGTTATGGCCGTTTA
miR-21-5p MI0000077	Synthetic miRNA		UAGCUUAUCAGACUGAUGUUGA
	Spec-TT	Primer RT	ATAAGCTACAACGACCAGAGCTAGAGAACCTAGCTCACCCACTACTCAACA
		PCR-forward	CATAAGCTACAACGACCAGAG

		PCR-reverse	GGTAGCTTATCAGACTGATGT
		Probe	AGAGAACCTAGCTCACCCACTAC
miR-146b-5p MI0003129	Synthetic miRNA		UGAGAACUGAAUUCCAUAGGCUG
	Spec-TT	Primer RT	CAGTTCTCCTATGCTCTCCAGGTACAGTTGGTACCTGTCTCCACTTCAGCCT
		PCR-forward	AGTTCTCCTATGCTCTCCA
		PCR-reverse	GAGAACTGAATTCCATAGGCT
		Probe	TACAGTTGGTACCTGTCTCCACTT
miR-20a-5p MI0000076	Synthetic miRNA		UAAAGUGCUUAUAGUGCAGGUAG
	Spec-TT	Primer RT	GCACTTTACGACGAATACTGCTAGAGTTGCTAGCAGAGCCCTTAACTACCT
		PCR-forward	GCGCACTTTACGACGAATAC
		PCR-reverse	CGTAAAGTGCTTATAGAGCAGGT
		Probe	AGAGTTGCTAGCAGAGCCCTTAA
miR-204-5p MI0000284	Synthetic miRNA		UUCCCUUUGUCAUCCUAUGCCU
	Spec-TT	Primer RT	GACAAAGGGATATGTGAGACGTACGTTGAGTACGTCAAGTGAAGTAGGCAT
		PCR-forward	GACAAAGGGATATGTGAGAC
		PCR-reverse	CCCTTGTCATCCATGCCT
		Probe	ACGTACGTTGAGTACGTCAAGTG
