# Supplementary figures and images for: Reciprocal Dysregulation of MiR-146b and MiR-451 Contributes in Malignant Phenotype of Follicular Thyroid Tumor
Source: Int J Mol Sci. 2020 Aug 19;21(17):5950. doi: 10.3390/ijms21175950 (PMC7503510; doi:10.3390/ijms21175950)

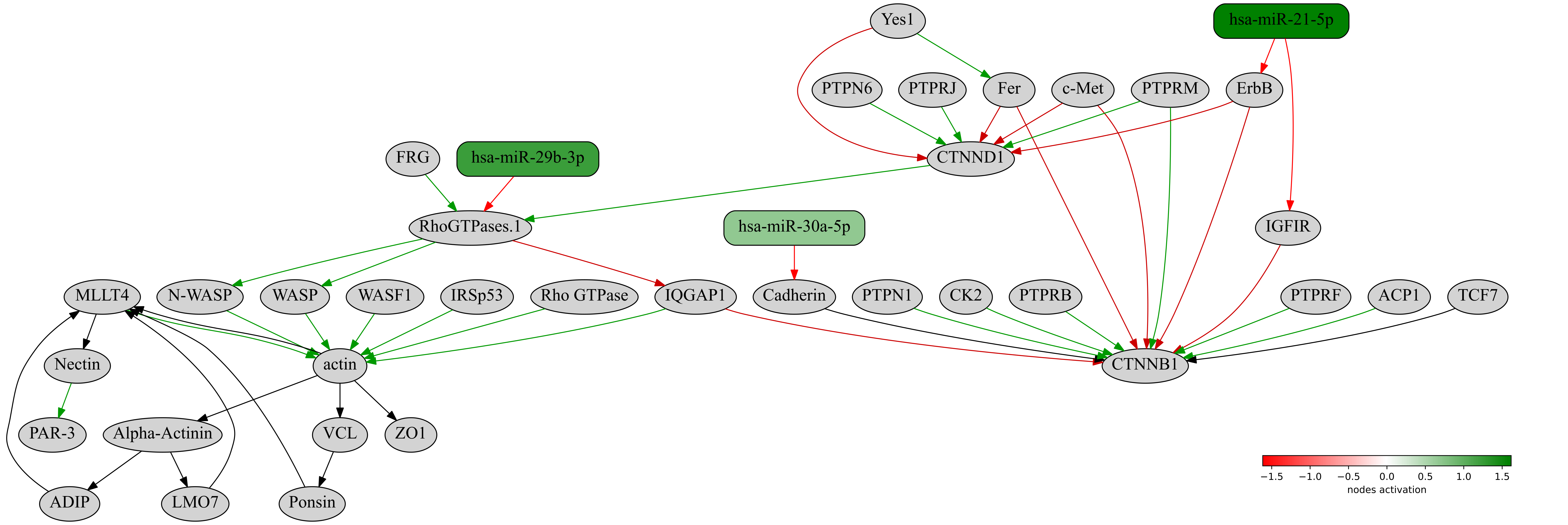

Supplement: Supplementary file 1 [file ijms-21-05950-s001.zip › Suppl 3.1. KEGG_Adherens_junction_Main_Pathway.png]

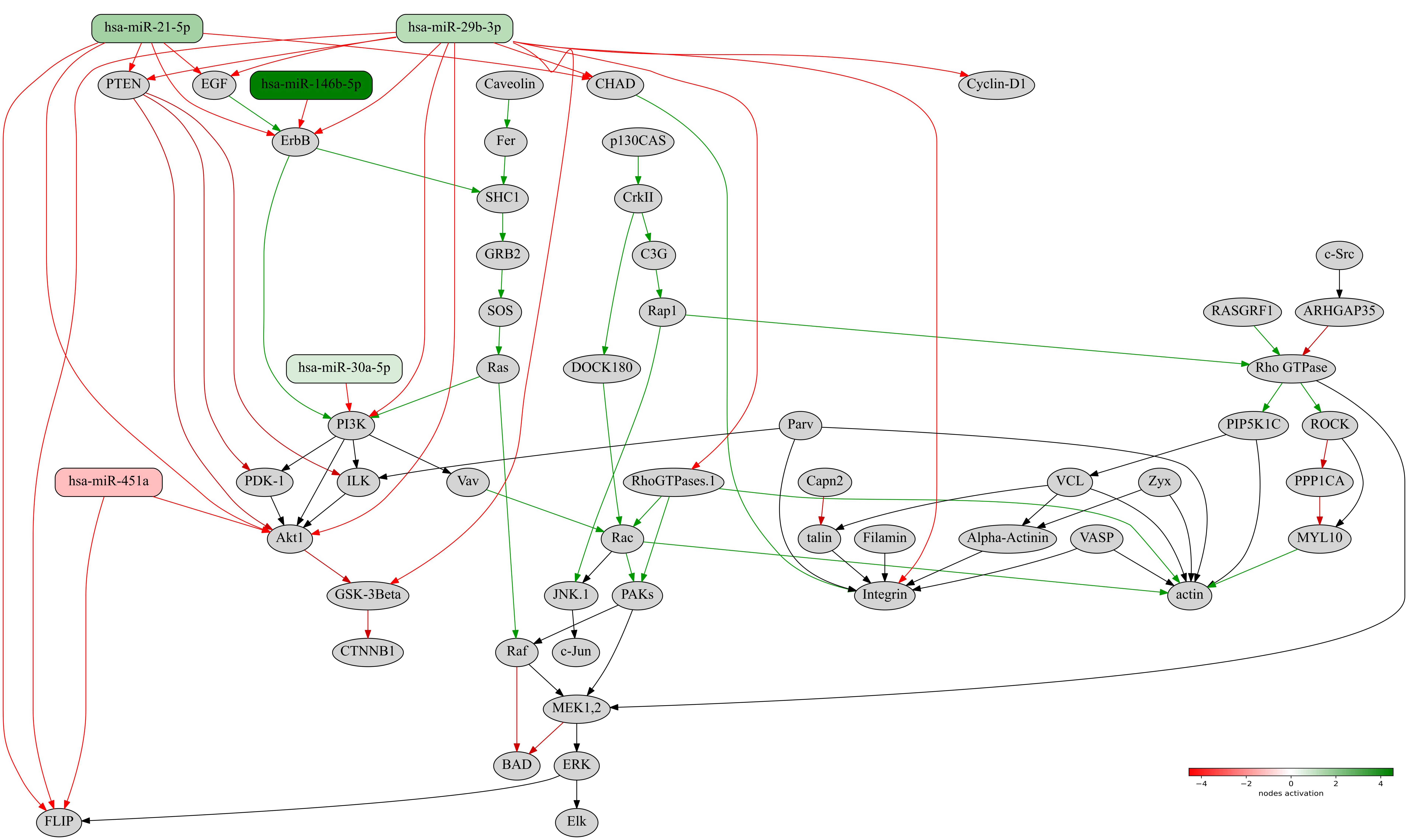

Supplement: Supplementary file 1 [file ijms-21-05950-s001.zip › Suppl 3.2. KEGG_Focal_adhesion_Main_Pathway.png]

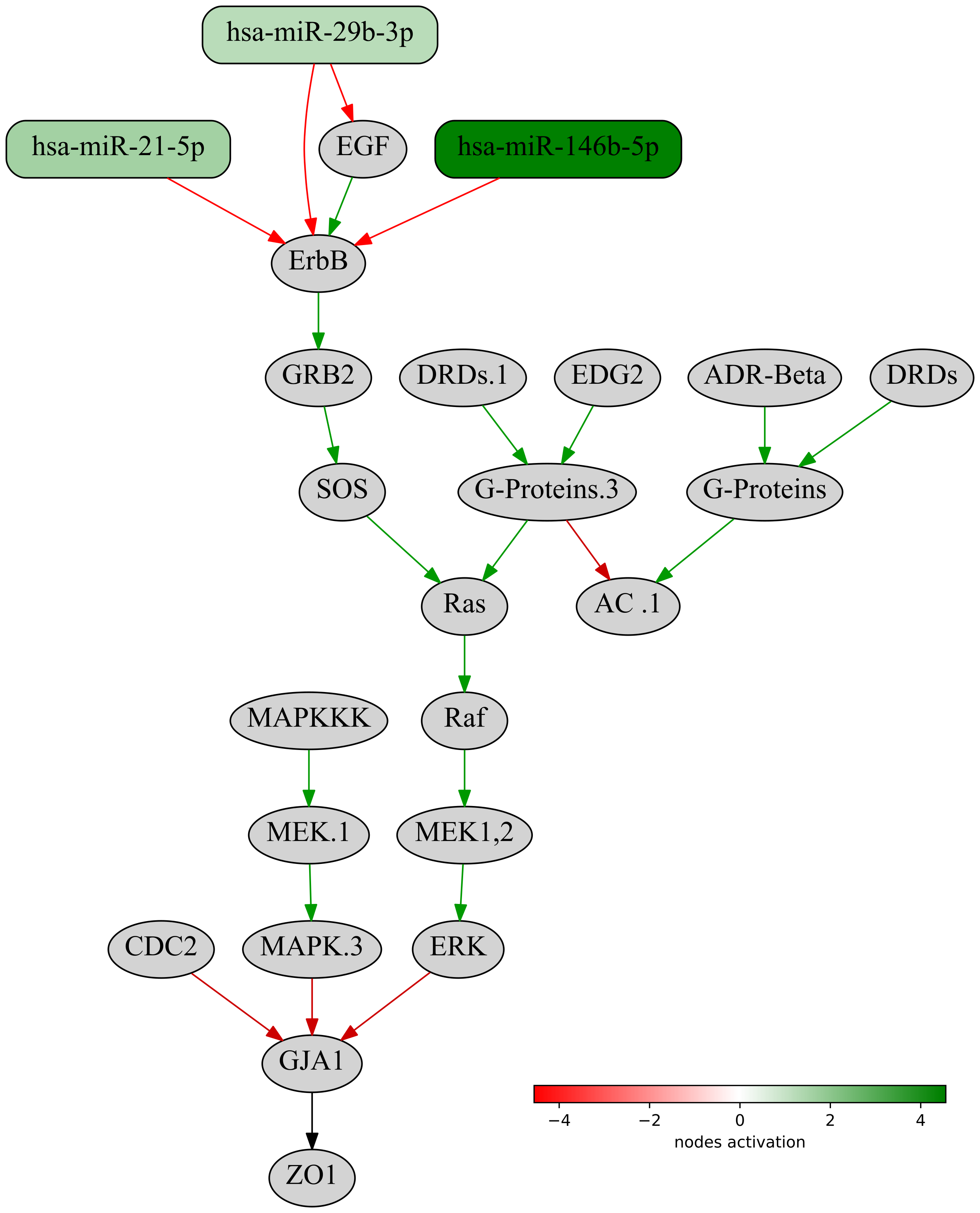

Supplement: Supplementary file 1 [file ijms-21-05950-s001.zip › Suppl 3.3. KEGG_Gap_junction_Main_Pathway.png]

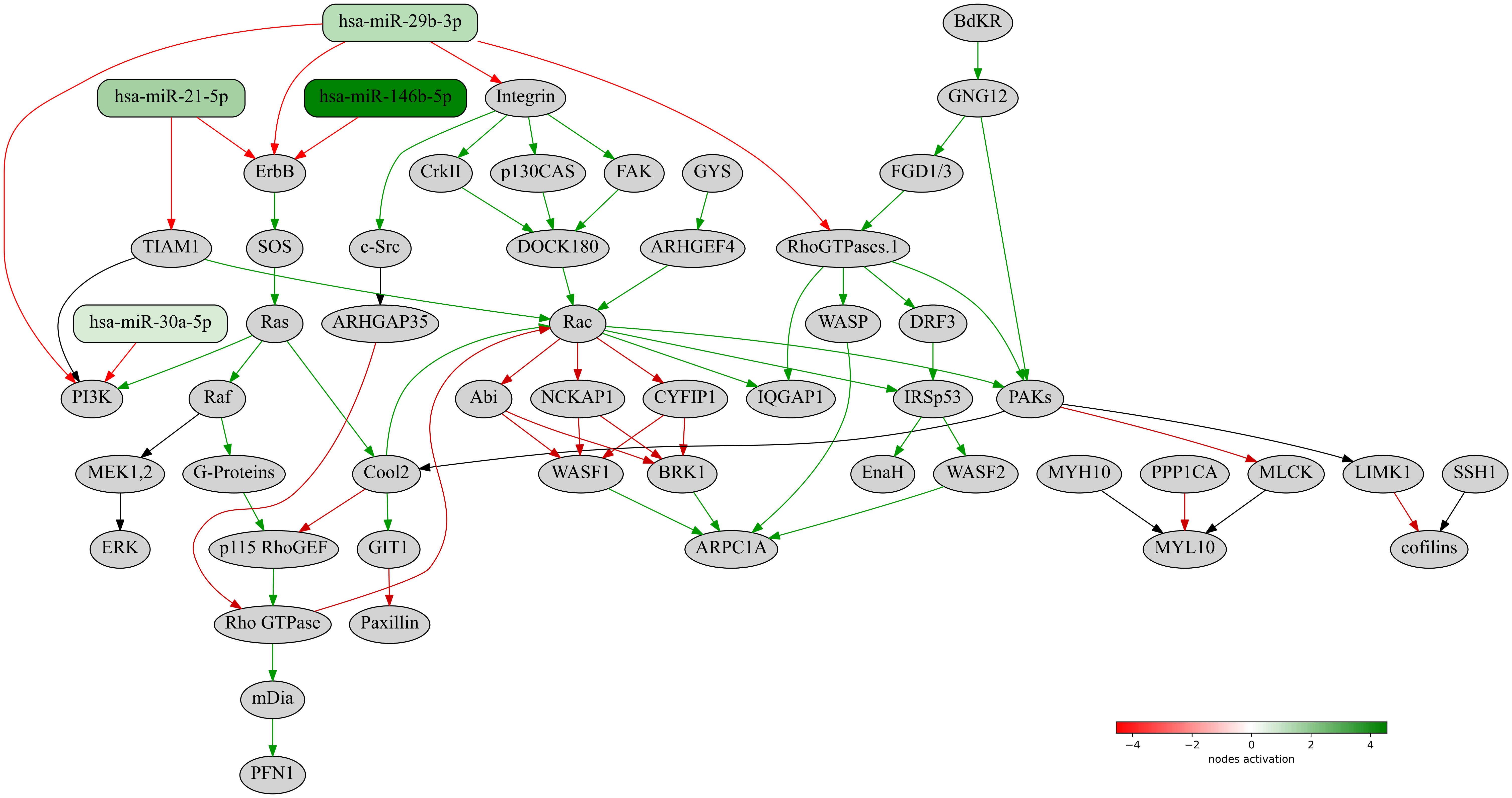

Supplement: Supplementary file 1 [file ijms-21-05950-s001.zip › Suppl 3.4. KEGG_Regulation_of_actin_cytoskeleton_Main_Pathway.png]

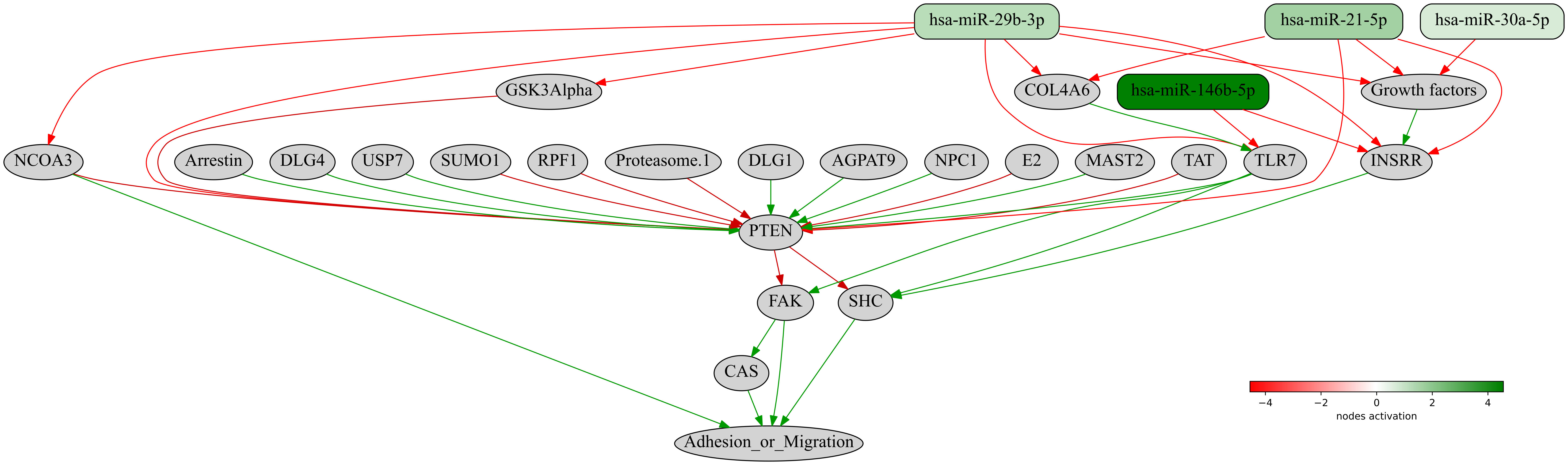

Supplement: Supplementary file 1 [file ijms-21-05950-s001.zip › Suppl 3.5. PTEN_Pathway_Adhesion_or_Migration.png]

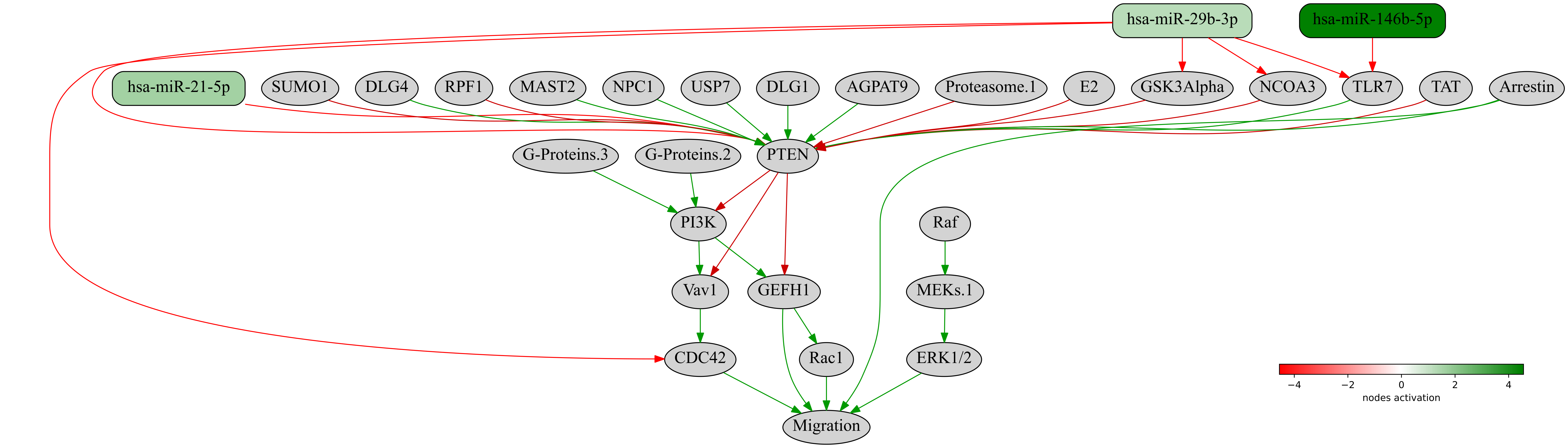

Supplement: Supplementary file 1 [file ijms-21-05950-s001.zip › Suppl 3.6. PTEN_Pathway_Migration.png]
